# Pancreatic and biliary obstruction years after retention of a swallowed coin in a duodenal diverticulum: a case report

**DOI:** 10.1186/s13256-015-0608-6

**Published:** 2015-06-18

**Authors:** Ibrahim Ismail, David W Mudge

**Affiliations:** 1 Cairns Hospital, 165-171 Esplanade, Cairns North, QLD Australia; 2 University of Queensland at Princess Alexandra Hospital, 199 Ipswich Road, Woolloongabba, QLD Australia

**Keywords:** Duodenal diverticulum, Pancreatitis, Foreign body, Endoscopic retrograde, Cholangiopancreatography, Coin

## Abstract

**Introduction:**

Congenital duodenal diverticula are a rare anomaly. The discovery of one in association with an ingested foreign body has only been reported on one previous occasion. In this challenging presentation, the presence of the coin led to the correct diagnosis. Patients with congenital duodenal anomalies may present a number of associated abnormalities. Interestingly, after the discovery of his intraluminal duodenal diverticulum, we searched and found that our patient presented a number of associated pathologies, as described in the literature.

**Case presentation:**

Our patient was a 36-year-old man, Caucasian, a kidney transplant recipient who presented with abdominal pain, vomiting and fever after an episode of pancreatitis. Because of a history of behavioral problems associated with intellectual impairment, including a compulsion to swallow coins during childhood, an abdominal radiograph was performed. Surprisingly, the radiograph revealed a radiopaque shadow in the central abdominal area. The findings of the ultrasound examination and computed tomography scan were suggestive of dilated biliary and pancreatic ducts. We performed an endoscopic retrograde cholangiopancreatography, which led to confirmation of the suspected coin above an obstructing intraluminal duodenal diverticulum with associated biliary ductal dilation. Upon retrieval of the coin, it was found to be a 1975 copper two-cent piece out of circulation in Australia for a large number of years.

**Conclusions:**

Foreign body retention in the gastrointestinal tract in an adult could be a sign of underlying mechanical pathology. Intraluminal duodenal diverticulitis can have a varied presentation, including life-threatening complications. Awareness should be raised of the conditions associated with congenital duodenal anomalies in adults, including renal, hepatobiliary and cardiac defects, many of which were present in our case.

## Introduction

Diverticula, sac-like protrusions of the intestinal wall, occur throughout the small and large intestines. The presence of these lesions should be considered in patients with unexplained gastrointestinal bleeding, intestinal obstruction, acute abdomen, chronic abdominal pain, anemia, or malabsorption [1]. The most frequent location of diverticula in the small intestine is the duodenum. Congenital duodenal diverticula are rare anomalies that usually remain asymptomatic. However, patients can present duodenal obstruction with post-cibal fullness or pain relieved by vomiting as the first manifestation of a duodenal diverticulum. Pancreatitis was the presenting manifestation of intraluminal duodenal diverticulum in 20% of the reported cases [2]. The pathogenesis of pancreatitis is also unclear in patients with intraluminal duodenal diverticulum. However, reflux of duodenal contents through the papilla of Vater is the most widely accepted cause [3,4]. Occlusion of the biliary or pancreatic duct from an enlarging diverticulum pouch has also been reported. We believe the former as the main cause of pancreatitis in our patient owing to the tight duodenal stenosis from the obstructing intraluminal diverticula with the entrapped coin that promoted duodenal content/bile reflux into the more patent pancreaticobiliary system. As far as we know, the discovery of a duodenal diverticulum in an adult patient in association with an ingested coin has only been reported once [5].

## Case presentation

A 36-year-old Caucasian man with a past history of intellectual impairment, epilepsy and end-stage kidney disease diagnosed in childhood, leading to kidney transplantation at age 14, presented to our hospital.

**Figure 1:**
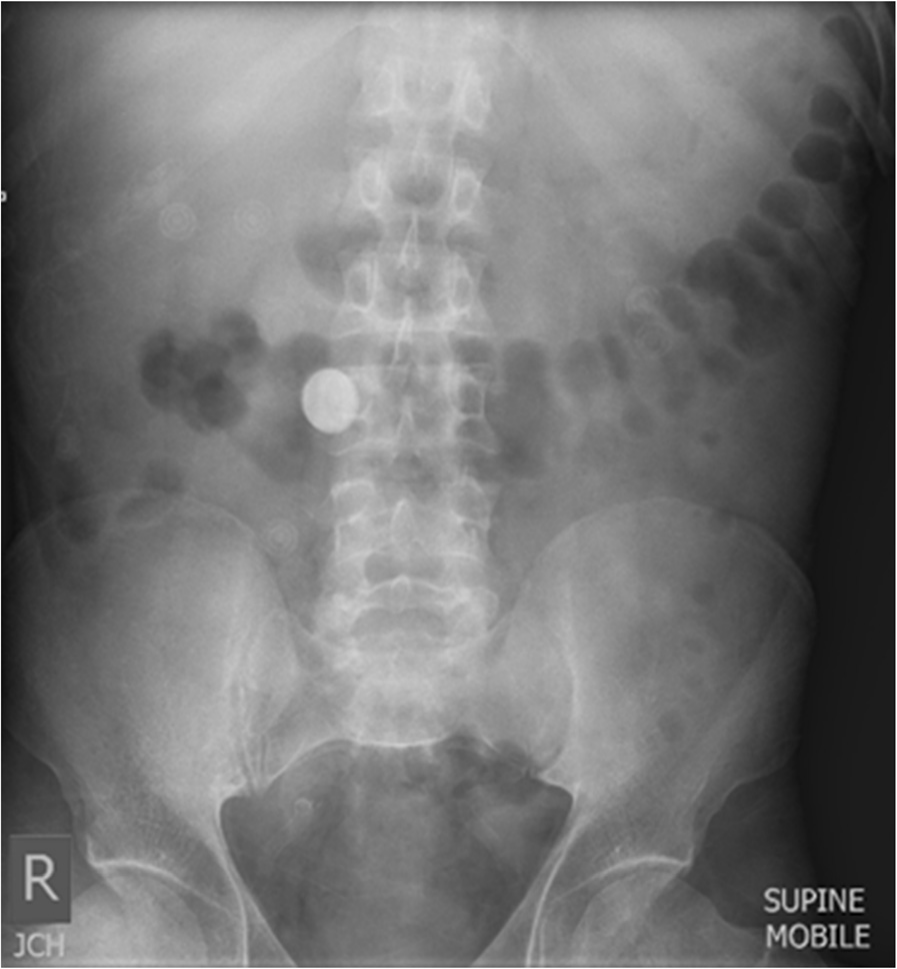
Plain abdominal radiographs showing the radiopaque coin at presentation.

**Figure 2:**
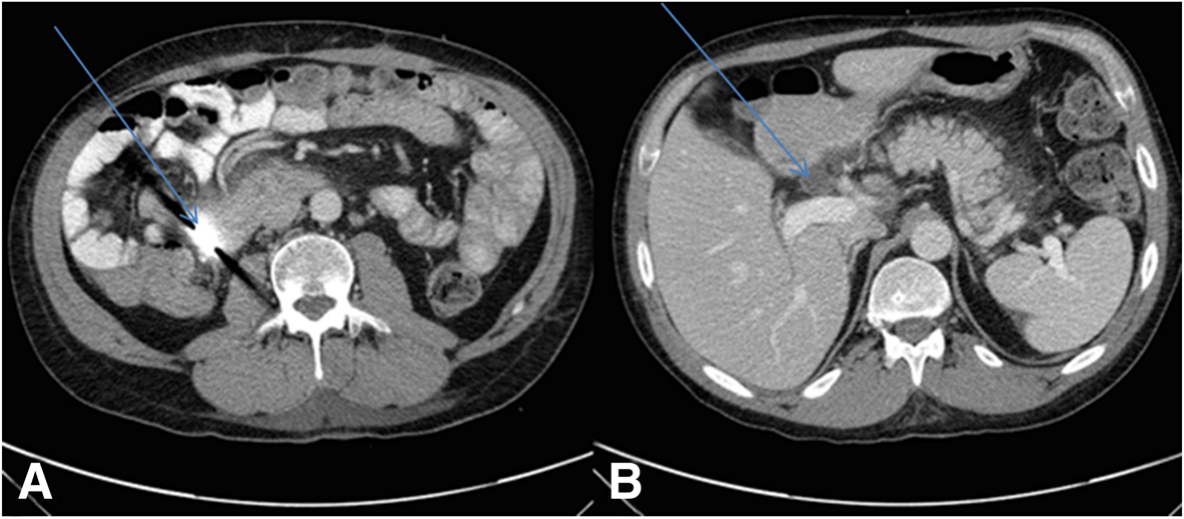
Computerised tomography scans with contrast at presentation. **(A)** Showing the coin (arrow). **(B)** Dilated common bile duct (arrow).

He had high-grade fever, abdominal pain, and had been vomiting for a week. While obtaining the medical history from his mother, she described a propensity to swallow coins related to childhood behavioral difficulties. Despite her suggestion that he had not done so since the age of 12, an abdominal radiograph was performed. Surprisingly, it revealed a periumbilical, circular, radiopaque density, thought to be a coin (Figure 1). However, the double-bubble sign or halo sign was absent. There were no clinical features of gastric outlet syndrome to suggest duodenal stenosis leading up to the presentation. A computed tomography scan with contrast localized the coin in the second part of the duodenum, revealing the suspected diverticula, and common bile duct and pancreatic duct dilatation (Figure 2). Blood laboratory tests suggested pancreatitis: bilirubin 14umol/L (<20), alkaline phosphatase (ALP) 132U/L (53 to 128), gamma glutamyl transferase (GGT) 269U/L (<55), alanine aminotransferase (ALT) 29U/L (<45), aspartate aminotransferase (AST) 11U/L (<35), and lipase –580U/L (<60).

After broadening the antibiotic coverage due to concerns regarding ascending cholangitis, our patient underwent an emergency endoscopic retrograde cholangiopancreatography, which confirmed not only the nature of the foreign body but also the underlying anatomical abnormality causing the duodenal and pancreaticobiliary dilatation. Both common bile duct and common hepatic duct were moderately dilated up to 15mm with no intrahepatic ductal dilatation. Even though no purulent bile was seen, due to concerns of ascending cholangitis, a common bile duct stent was inserted during the procedure following removal of the coin.

**Figure 3:**
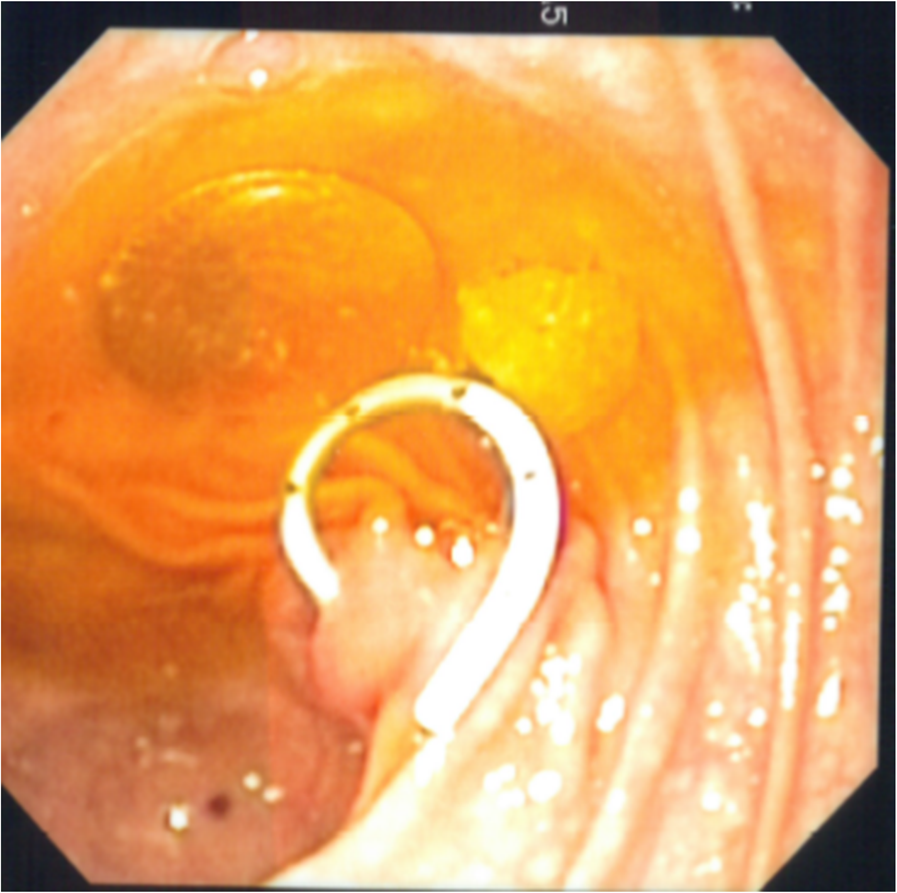
The coin is seen with a pigtail catheter in this image taken during the endoscopic retrograde cholangiopancreatography to investigate suspected cholangitis.

When retrieved, the coin was found to be a 1975, copper two-cent piece (Figure 3). Such coins have been out of circulation in Australia since 1994, suggesting that the coin may have been swallowed and lodged in the duodenal diverticulum at least 14 years earlier, a hypothesis consistent with the collateral history obtained from the patient’s mother.

The differential diagnosis of duodenal stenosis could be broadly classified into congenital and acquired causes. Congenital obstruction is caused by either an intrinsic or extrinsic anomaly. Intraluminal duodenal diverticulum and duodenal atresia comprise intrinsic stenoses. Annular pancreas, choledochal cyst, and malrotation account for most extrinsic obstructions [6]. All of these differential diagnoses were effectively excluded by the investigations instituted.

Our patient underwent an elective reconstruction of the obstructed duodenum by Roux-en-Y. During surgery, we confirmed the tight stricture at D2 distal to the pancreas with proximal dilatation of the duodenum and significantly dilated bile duct.

## Discussion

Small bowel diverticula occur most frequently in the duodenum. Intraluminal diverticula are usually congenital, whereas the acquired diverticula are mostly extraluminal. Cases of acquired diverticula account for the vast majority of duodenal diverticula. These acquired diverticula should be distinguished from congenital intraluminal duodenal diverticula, which are often associated with other abdominal abnormalities, especially affecting the pancreatic and biliary anatomy [7]. In fact, more than 50% of the affected patients have associated congenital anomalies, including pancreatic anomalies, intestinal malrotation, esophageal atresia, Meckel’s diverticulum, variants of imperforate anus, congenital heart disease, central nervous system lesion, renal anomalies and rarely, biliary tract anomalies [8,9]. Moreover, the association of congenital intraluminal duodenal diverticula with Down’s syndrome is well documented [10]. In our patient, it was interesting to note the previously existing renal failure and intellectual impairment, in addition to the diagnosis of ascending aortic aneurysm many months after this presentation. Apart from pancreatitis, serious complications may include hemorrhage from ulceration within the diverticula [11] and cholangitis [12].

Foreign body ingestion is a common problem presenting at the emergency departments. The majority of the ingested foreign bodies pass through the gastrointestinal tract spontaneously. More than 90% of the impacted foreign bodies are amenable to endoscopic retrieval. Ninety percent of the foreign bodies that reach the stomach will undergo passage. After the initial suspicion of a more sinister pathology such as malignancy in our patient, it was a relief to find that the culprit was a coin. In fact, it was a 1975 two-cent coin. Whether the coin contributed to our patient’s clinical presentation is unclear, but it was certainly helpful for establishing the diagnosis in this challenging presentation. The Australian two-cent coin was introduced in 1966, and it was withdrawn from circulation in 1994. We hypothesize that the coin lodging in the duodenal diverticulum led to progressive duodenal stenosis over a long period of time, although we concede that this is difficult to prove. Neither our patient nor his mother were collectors of old coins and they denied having recent access to them, which supports the theory of the remote swallowing. It is possible that the duodenal stenosis developed independently of the presence of the coin, but it is unusual that it could have been asymptomatic for a long period of time and not lead to an earlier presentation.

## Conclusions

Foreign body retention in the gastrointestinal tract in adults could be a sign of underlying mechanical pathology as illustrated above. To our knowledge, this is the first case to describe a duodenal diverticulum associated with an entrapped coin presenting with pancreatitis. Additionally, this case should raise awareness of the conditions associated with congenital duodenal anomalies in adults, including renal, hepatobiliary and cardiac defects that could be life-threatening for these patients.

## Consent

Written informed consent was obtained from the patient’s guardian for publication of this case report and accompanying images. A copy of the written consent is available for review by the Editor-in-Chief of this journal.

## Abbreviations

ALP: alkaline phosphatase
ALT: alanine aminotransferase
AST: aspartate aminotransferase
GGT: gamma glutamyl transferase
